# Radiomic Analysis as a Powerful Tool for Cytological Images of Benign Thyroid Nodules Treated by Thermal Radiofrequency Ablation

**DOI:** 10.3390/bioengineering13020171

**Published:** 2026-01-30

**Authors:** Alessia Finti, Franco Marinozzi, Michela Franzò, Flavia Federici, Matteo Bolognese, Alessandro Giusti, Andrea Leoncini, Fabiano Bini

**Affiliations:** 1 Department of Mechanical and Aerospace Engineering, Sapienza University of Rome, 00184 Rome, Italy; alessia.finti@uniroma1.it (A.F.); franco.marinozzi@uniroma1.it (F.M.); federici.1584729@studenti.uniroma1.it (F.F.); bolognese.1813797@studenti.uniroma1.it (M.B.); 2 Institute of Bioimaging and Complex Biological Systems, National Research Council (IBSBC-CNR), Contrada, Pietrapollastra-Pisciotto, 90015 Cefalù, Italy; 3 Department of Psychology, Sapienza University of Rome, 00184 Rome, Italy; michela.franzo@uniroma1.it; 4 Dalle Molle Institute for Artificial Intelligence, USI-SUPSI, 6962 Lugano, Switzerland; alessandro.giusti@supsi.ch; 5 Integrated Diagnostics of Italian Switzerland Institute (IDISI), Radiology Clinic, Ente Ospedaliero Cantonale, 6900 Lugano, Switzerland; andrea.leoncini@eoc.ch

**Keywords:** radiofrequency ablation (RFA), benign thyroid nodules, radiomics, cytological analysis

## Abstract

(1) Background: Thermal radiofrequency ablation (RFA) is an innovative treatment for benign thyroid nodules. This study aims to identify morphological and texture-based cytological parameters through radiomic and cytological analysis of fine-needle aspiration cytology (FNAC) images to support the prediction of the nodules’ response to RFA. (2) Methods: The study, conducted in collaboration with EOC—Ente Ospedaliero Cantonale (Lugano, Switzerland), analyzed FNAC images from three patients with benign thyroid nodules treated with RFA. Radiomic features were extracted in PyRadiomics and analyzed through Principal Component Analysis (PCA). A MATLAB (R2024b)-based workflow was implemented for automated chromatic and morphological analysis. (3) Results: Chromatic Analysis correctly identified separated nuclei with approximately 5% remaining unrecognized. Radiomics revealed robust connections between nuclear shape descriptors and texture-based features, showing the potential of a combined morphological-radiomic approach. PCA indicated that texture and first order features played a significant role in cytological heterogeneity. (4) Conclusions: A combination between radiomics, chromatic, and morphological analysis provides a deeper understanding of thyroid nodule characteristics. By capturing texture and intensity variations often missed by traditional methods, radiomics may enhance prediction of post-RFA behavior. The proposed methodology provides a foundation for predictive models of Volume Reduction Ratio (VRR), improving personalized diagnosis, treatment planning, and follow-up.

## 1. Introduction

Thyroid nodules are a common clinical problem with significant impacts on patient health. From a diagnostic point of view, fine-needle aspiration cytology (FNAC) should be considered as the most effective preoperative diagnostic technique in identifying thyroid nodules [1]. According to the recommendations of Korean guidelines, thyroid nodules should be identified as benign based on at least two US-guided fine-needle aspirations (FNAs) or core needle biopsies (CNBs) before performing radiofrequency thermal ablation (RFA) [2] to exclude a false positive diagnosis of follicular neoplasms. The Bethesda System for Reporting Thyroid Cytopathology (TBSRTC) is shared worldwide as the reporting system for thyroid cytopathology, allowing the identification of diagnostic categories in terms of neoplastic risk and management strategies [3]. However, despite FNAC being considered the gold standard [4,5] for preoperative diagnosis, its interpretation and application vary among operators, institutions, and countries, highlighting the need for more objective and reproducible approaches. Moreover, variability in treatment responses highlights the need for personalized approaches.

RFA [6] is an innovative minimally invasive technique for the treatment of benign thyroid nodules. In recent years, non-surgical treatments for benign thyroid nodules have gained increased attention due to the possibility of reducing nodular volume without surgery. RFA is typically performed under local anesthesia to reduce the size of benign thyroid nodules and uses heat generated by radio waves to create thermal damage to the nodule tissue [7,8,9,10]. The entire procedure is performed under ultrasound guidance to ensure complete control over the procedure and maximum respect for the structures surrounding the nodule. A thin electrode needle (18–19 G) [11] is inserted by the specialist doctor through the skin of the neck and through the isthmus into the target tissue. Once the needle electrode has been correctly positioned, high-frequency energy is delivered through the active tip of the needle electrode, which generates heat and creates thermal damage to the nodular tissue. Radiofrequency (RF) energy allows for obtaining coagulative tissue necrosis through a process known as ‘thermo-ablation’. The needle electrode of the RF generator generates a high-frequency current flow, producing coagulative necrosis of the nodular tissue through the Joule effect. Several studies have also explored optimization strategies for RF ablation parameters in order to achieve the target volume reduction for a thyroid nodule [12]. In addition, Previous studies on thermal ablation mechanisms and energy–tissue interactions provide relevant methodological background for radiofrequency-based treatments [13].

In this context, advanced computational techniques for cytological image analysis are emerging. Digital Pathology, based on Whole Slide Images (WSI) analysis of histological or cytological specimens, enables the development of automated pipelines for quantitative measurements and computer-aided diagnosis (CAD) systems [14]. Automation of image classification and cellular detection using computer vision techniques facilitates high-throughput analysis of complex microscopy images, particularly in multiplexed microscopy [15]. Such techniques aim to improve diagnostic objectivity and reproducibility of expert assessments. Previous studies have demonstrated the utility of automated segmentation algorithms and nuclear morphological analysis to extract quantitative parameters such as area, circularity, and eccentricity [16,17]. Quantitative morphological parameters, such as area, eccentricity, and chromatin density, have been shown to improve the distinction between benign and suspicious cells [18]. Beyond segmentation and classification of cytology images, the integration of radiomics with deep learning techniques has shown promising results in identifying morphological patterns associated with neoplastic risk, providing objective support for diagnosis [19]. Radiomics is an advanced image analysis technique that enables the extraction of numerous quantitative features from medical images, providing additional information that may not be discernible through conventional visual assessment. This approach has been successfully applied in cytological image classification using deep learning and PCA-based classifiers to enhance diagnostic accuracy [20]. In the study regarding prognosis prediction in hepatocellular carcinoma, the concept of Pathomics is introduced, which integrates radiomics with pathological data, improving the predictive process [21]. The use of radiomics for segmentation and classification of nuclei from Whole-Slide Images (WSI) shows how radiomic features can differentiate nuclear variants more accurately than traditional segmentation [22]. In contrast, the study by Linares et al. [23] proposes a radiomics-based framework for histological classification of patients with interstitial lung disease, and radiomic features were extracted from cytological images of benign thyroid nodules to complement the morphological analysis.

In the emerging context of radiomics applied to histological images, the current study explores the potential of radiomic, chromatic, and morphological histological image analysis to identify morphological and texture-based parameters that could serve as key parameters for predicting the response of benign thyroid nodules to RFA treatment. While radiomics has been widely applied to the diagnosis and characterization of thyroid nodules through different imaging modalities, such as ultrasounds [24,25], computed tomography [26], and PET/CT [27], its application on histological images remains unexplored. The aim of this study is to validate radiomics as a powerful tool for thyroid nodules characterization, laying the foundation for the creation of a predictive model that, starting from the radiomic analysis of cellularity, can provide the doctor with information on the response of the thyroid nodule (VRR) to RFA. Given the limited sample size, this work should be interpreted as an exploratory methodological study rather than an attempt to establish a direct clinical association between cytological radiomics and VRR. Instead, the goal is to generate hypotheses and provide a structured analytical framework to support future studies with larger cohorts.

## 2. Materials and Methods

### 2.1. Study Design and Data Collection

The current study was conducted in collaboration with EOC—Ente Ospedaliero Cantonale (Lugano, Switzerland), involving cytological and related ultrasound images and reports of three patients (A, B, and C) with symptomatic benign thyroid nodules treated with RFA at the Radiology and Diagnostic department at EOC. For these patients, in addition to cytological images, thyroid ultrasound reports before the RFA treatment and one month and six months after the RFA procedure were available (except for Patient C, who did not attend the six-month follow-up).

### 2.2. Diagnostic and Therapeutic Procedures

#### 2.2.1. Fine-Needle Aspiration Cytology (FNAC)

Fine-Needle Aspiration Cytology (FNAC) [4,5] is a gold-standard, non-invasive diagnostic procedure used to confirm or exclude the malignant nature of a thyroid nodule. FNAC involves sampling cells using a fine needle (20–25 G) inserted into the nodule through the thyroid. The entire FNAC procedure is conducted under ultrasound guidance to improve its accuracy.

The cell sample is analyzed under a microscope by a professional who provides the final diagnosis on the nature of the nodule.

#### 2.2.2. Thyroid Ultrasound (ECO) Parameters

Thyroid ultrasound is a low-cost, non-invasive imaging procedure that allows the evaluation of the morphological characteristics of the thyroid nodule and surrounding structures. In the treatment of thyroid nodules, ultrasound plays a fundamental role in the primary evaluation of morphological characteristics, during the RFA procedure, and in the follow-up period. Unlike other diagnostic imaging techniques, ultrasound images are displayed and analyzed in real-time, enabling immediate evaluation of the nodules’ characteristics, such as nodular volume. Typically, thyroid nodules are ellipsoidal, allowing the use of the ellipsoid formula, represented in Equation (1), to calculate nodular volume [28,29], where V is the volume in milliliters (mL) or cubic millimeters (mm^3^), and d1, d2, and d3 are the three spatial diameters of the nodule.

(1) V = 0.5 ∙ d1∙d2∙d3

This measurement is essential for both the initial evaluation and the success assessment of **RFA** treatment in benign thyroid nodules. The Volume Reduction Ratio (**VRR**) formula [7,29] is described in Equation (2).

(2) $$VRR=\frac{\left(Initial\;volume-volume\;post\;RFA\right)}{initial\;volume}\times 100$$

In the current study, the post-treatment volume (volume post **RFA**) was calculated at 1 month (1st follow-up) and 6 months after the procedure (2nd follow-up).

#### 2.2.3. RFA Procedure—VIVA RF System

RFA was performed under local anesthesia using the VIVA RF System (STARmed Co. Ltd., Goyang, Republic of Korea), a radiofrequency ablation system that allows the treatment of pathological tissues such as benign thyroid nodules. The VIVA RF SYSTEM consists of an RF generator and a peristaltic pump. The generator uses radiofrequency energy to generate coagulative tissue necrosis. The peristaltic pump, needle electrodes, and dispersion electrodes constitute a system that allows tissue ablation. The system creates necrosis of the target tissue by passing high-frequency current between the needle electrode and the dispersion electrode. The current encounters a resistance (impedance) from the tumor tissue, which, therefore, due to the Joule effect, generates a local increase in temperature.

RFA procedures reported in this study were performed by experienced interventional radiologists according to the international standards for the ablation of benign thyroid nodules.

### 2.3. Cytology Image Processing and Feature Extraction

#### 2.3.1. Image Acquisition

**Figure 1:**
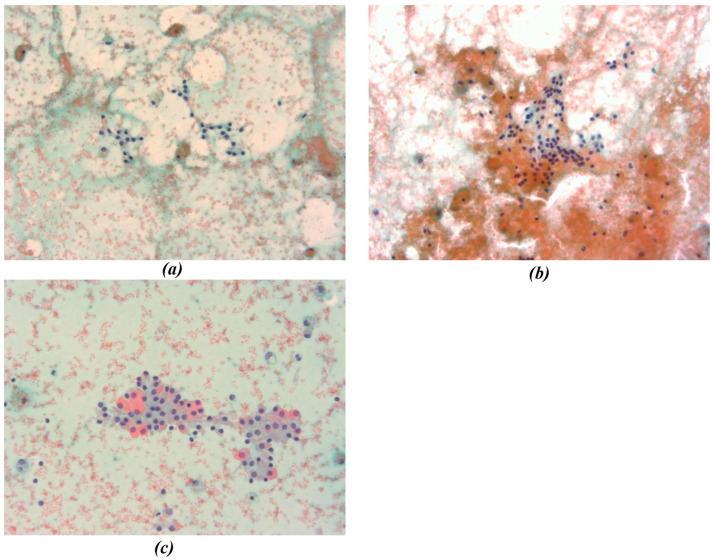
(**a**–**c**) Representative cytological images from Patient A. All smears were fixed and stained using the Papanicolaou technique and acquired at 40× magnification. Images show thyroid follicular cell nuclei used for chromatic segmentation and radiomic feature extraction at high magnification. In the figure the different chromatic shades were obtained by applying the Papanicolaou technique.

**Figure 2:**
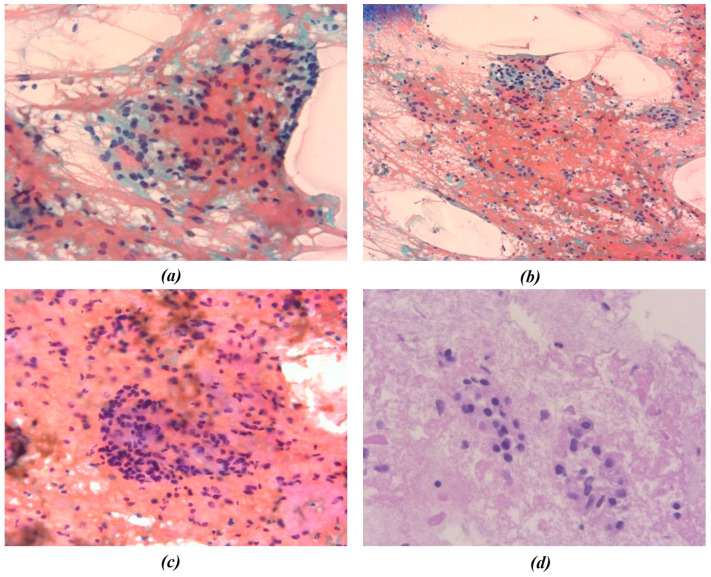
Representative cytological images from Patient B. (**a**–**c**) Papanicolaou-stained smears; (**d**) hematoxylin and eosin (H&E) staining. Images were acquired at different magnification levels (10× and 40×) and were independently processed for chromatic segmentation and radiomic feature extraction to evaluate the effect of magnification on feature variability. The figures show the different chromatic shades obtained by applying the procedures mentioned.

**Figure 3:**
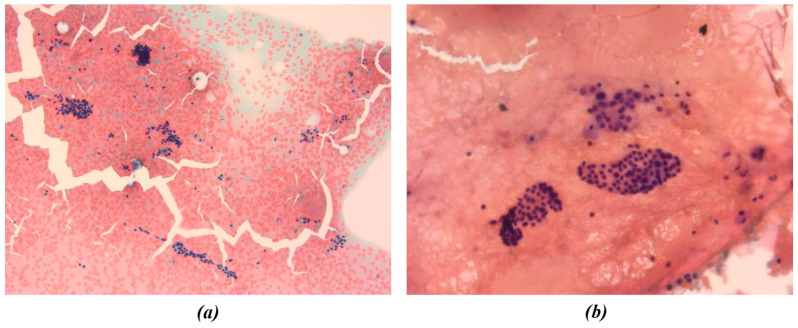
Representative cytological images from Patient C. All smears were fixed and stained using the Papanicolaou technique. Images were acquired at 10× (**a**) and 40× (**b**) magnification and were both included in the radiomic and morphological analysis pipelines. The figures show the different chromatic shades obtained by applying the above-mentioned procedure.

In this study, all cytological smears were fixed and stained using the Papanicolaou technique, except for the fourth image from the second cyto-block (Figure 2d), which was stained using Haematoxylin and Eosin (H&E) to allow for detailed histological examination. The magnification levels for the images vary among the patients according to the pathologist’s protocol of data acquisition. For Patient A, all images were consistently taken at 40× magnification (Figure 1a–c), ensuring high-resolution details across all samples. Patient B’s samples include a mix of magnifications, with the first image at 10× magnification (Figure 2a) to present an initial general view, followed by subsequent images at 40× magnification to focus on finer cellular details (Figure 2b–d). For Patient C, the first image was captured at 10× magnification (Figure 3a), providing a broader overview, while the second image (Figure 3b) was taken at 40× magnification to offer more detailed cellular morphology. This methodological approach allows for a comprehensive analysis of cytological and histological features across different magnification levels, but also requires a differentiated analysis for the two levels of magnification.

#### 2.3.2. Chromatic Analysis of Cytological Images

Given the importance of different color shades of thyroid nodules compared to the surrounding tissue, a chromatic analysis was performed in MATLAB on nuclei images through a segmentation process. Prior to segmentation, a Median filter was applied to the nine images to reduce “salt and pepper” noise for improving image quality by removing artifacts. Then, as reported in [30], the original images were first converted from RGB to Lab color space for an enhanced perception of the color spectrum. Secondly, the K-means clustering algorithm was employed to group image data into K clusters according to similarity criteria. The choice of K value was image-based: the optimization of cluster number was empirically evaluated to adapt segmentation to chromatic variability among samples. Then, the image was binarized to convert the segmented image into a nuclei map and to separate the ROIs from the remaining tissue.

To correctly identify separated nuclei in K-means segmentation masks, a further segmentation technique was employed: the Cellpose model for cell segmentation [31]. MATLAB Medical Imaging Toolbox’s Cellpose cyto2 model was used to segment nuclei through the segmentCells2D function. The algorithm labels each instance separately, as indicated by the different resulting-colored masks. Algorithm parameters, such as Flow Error Threshold and Cell Probability Threshold, were accurately chosen for each image to improve nuclei recognition and separation and to avoid over-segmentation. Eventually, the Cell Diameter parameter was chosen through measurement of the nuclei’ mean diameter in MATLAB’s Image Viewer tool.

#### 2.3.3. Radiomics Analysis and Principal Component Analysis (PCA)

Radiomic features of segmented nuclei were extracted through PyRadiomics (v. 3.0.1.) to ensure standardization and reproducibility in radiomic feature extraction. Pyradiomics is an open-source Python (v. 3.10.14.) library specifically developed for high-throughput radiomic analysis [32]. PyRadiomics adheres to the guidelines set by the Imaging Biomarker Standardization Initiative (IBSI), which promotes the standardization of radiomic feature definitions to enhance comparability across different studies and imaging modalities [33]. The radiomic features were extracted by applying PyRadiomics to segmented cytological images obtained through K-means and Cellpose techniques, using binary masks to define Regions of Interest (ROIs). The images and masks, processed in MATLAB, were exported and subsequently analyzed using PyRadiomics for radiomic feature extraction.

Radiomic feature extraction was performed in compliance with the Imaging Biomarker Standardization Initiative (IBSI) guidelines, considering predefined feature classes (shape2D, first-order statistics, and texture features). Radiomic feature extraction was performed using PyRadiomics with default parameter settings. No image intensity normalization or spatial resampling was applied to preserve the original chromatic and intensity distributions of the cytological images.

The extracted features were categorized into three main groups [33]. The first category, Shape2D features, quantifies geometric properties of the nuclei, including area, perimeter, major and minor axis length, and convexity. These features are essential in assessing structural variations in cells. The second category, Texture features, captures spatial patterns in pixel intensities within the segmented regions. The Gray-Level Co-occurrence Matrix (GLCM) and Gray-Level Run-Length Matrix (GLRLM) were used to extract features related to homogeneity, contrast, and entropy, offering insights into nuclear heterogeneity. The final category, First-Order Statistics (intensity features), describes the distribution of pixel intensity values within the nucleus, including metrics such as mean intensity, standard deviation, skewness, and kurtosis, which help quantify variability in cell composition.

Eventually, to reduce the dimensionality of the dataset and to identify the main features contributing to the variability in cytological samples, Principal Component Analysis (PCA) was applied. This technique was employed to assess the relationships between radiomic features and identify the principal components (PCs) that explain most of the variance within the dataset. The analysis was also conducted to explore the variance structure of radiomic features and identify those contributing most to data variability, with the perspective of informing future hypothesis-driven studies.

#### 2.3.4. Nuclei Shape Analysis

After chromatic segmentation and radiomics analysis, a morphological analysis was executed to make a comparison with radiomic 2D shape features and to identify nuclei shape parameters of interest for pathologists. The connected component analysis was implemented in MATLAB through MATLAB’s bwlabel and regionprops functions. To ensure the accurate separation of analyzed nuclear regions, outliers were removed based on the interquartile range (IQR), specifically within the 25th to 75th percentile range of mean area values for each image.

The shape analysis and measurement techniques, as described by Wirth [34], consists of computing parameters such as area, perimeter, length of the major and minor axes, and the area of the convex hull. However, in accordance with the pathologists’ request, the analysis focused on derived shape features (solidity, eccentricity, and circularity) calculated as described in [35].

Eventually, a correlation was studied between shape parameters and extracted radiomic shape features.

Given the exploratory and methodological nature of the study and the limited number of patients, no formal sample size calculation was performed. Individual nuclei were treated as image-derived observations and not as statistically independent samples.

Accordingly, no inferential statistical analysis or hypothesis testing was conducted; analyses were limited to descriptive statistics, correlation analysis, and exploratory multivariate techniques. No predictive model, classification task, or validation strategy was implemented, as the study was designed as an exploratory methodological analysis.

## 3. Results

### 3.1. Chromatic Analysis and Nuclei Segmentation

**Figure 4:**
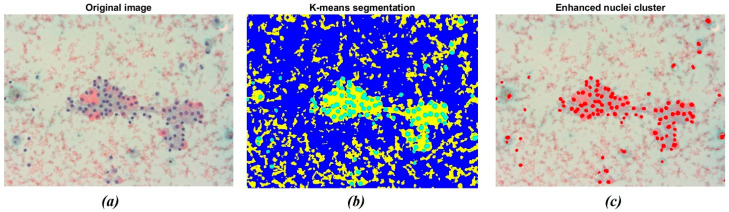
Example of nuclei segmentation with the K-means clustering method. (**a**) Original image showing a darker shade for nuclei tissue; (**b**) The result of segmentation using the K-means clustering method, which separates regions based on color difference; (**c**) The final segmentation of nuclei, highlighting the detected nuclei in red for further analysis.

The result of the application of the K-means clustering algorithm is shown in Figure 4. As stated before, the algorithm allowed grouping image data into *K* clusters according to similarity criteria (Figure 4b). Then, the image was binarized to convert the segmented image into a nuclei map and to separate the ROIs from the remaining tissue. The overlapping of the original image with the selected nuclei cluster is shown in Figure 4c. The resulting image consists of pixels belonging to a chromatic cluster, allowing for a deeper visualization of the sample’s dominating structures.

**Figure 5:**
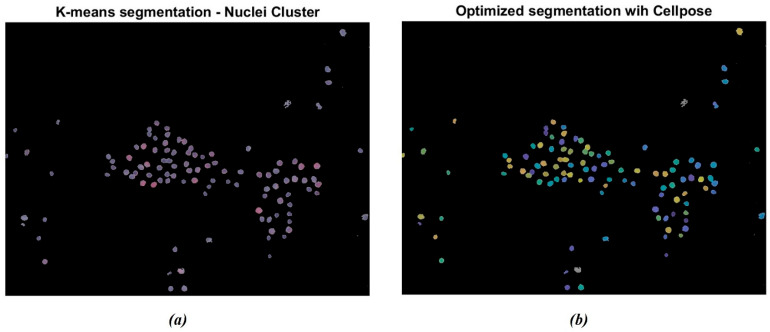
Example of nuclei segmentation with the Cellpose Cyto2 model. (**a**) Original cluster of isolated nuclei through K-means segmentation; (**b**) segmentation of nuclei through the Cellpose model: the algorithm labels each instance separately. The different chromatic shades show how many nuclei have been detected.

The segmentation was further enhanced through the Cellpose R2023b library tools in MATLAB. The algorithm labels each instance separately, as indicated by the different colored masks in Figure 5. Figure 5a represents the original cluster of isolated nuclei through the K-means algorithm. It is possible to observe that some nuclei appear to be segmented as paired structures and not correctly separated. Otherwise, as reported in Figure 5b, the Cellpose algorithm allowed for the separation of the paired nuclei, enhanced through different color shades. The gray-colored nuclei, consisting of approximately 5% of the total amount of cells, represent the nuclei that were not actually recognized by the Cellpose algorithm. This percentage remained uniform across the dataset.

### 3.2. Volume Reduction Ratio (VRR) and Shape Analysis

Using ultrasound, it was possible to evaluate the volume of each nodule before the RFA treatment, one month after the RFA (first follow-up) treatment, and 6 months later (second follow-up). The VRR for each patient was calculated by applying the VRR formula (Equation (2)). This enabled the evaluation of the possible success of the treatment in reducing the size of the thyroid nodule. Table 1 presents the volume of each patient’s nodule before RFA treatment, 30 days after, and 6 months later, as well as the respective calculated VRR.

VRR results indicate that all patients experienced a reduction in nodule volume at the first follow-up (30 days). At six months, volume changes varied among patients, with some showing further reduction and others slight increases. This increase aligns with the widespread phenomenon of growth of thyroid nodules after thermo-ablation treatment with radio frequencies (RFA), which can be attributed to several factors [36,37,38].

Nuclei morphological parameters were computed to make a comparison between radiomic 2D shape features and shape analysis for thyroid nodules analysis. Table 2 shows the Average and Standard Deviation (Std) of the morphological parameters (solidity, circularity, and eccentricity) that characterize the nuclei of each patient. The specific reported parameters were extracted in accordance with the pathologists’ request.

Since the analyzed dataset consists of images taken at different levels of magnification, they were analyzed separately. Thus, only the results for images acquired at 40× magnification, which account for seven out of the nine images analyzed, are reported in the mentioned table.

### 3.3. Radiomics and PCA Outcomes

**Figure 6:**
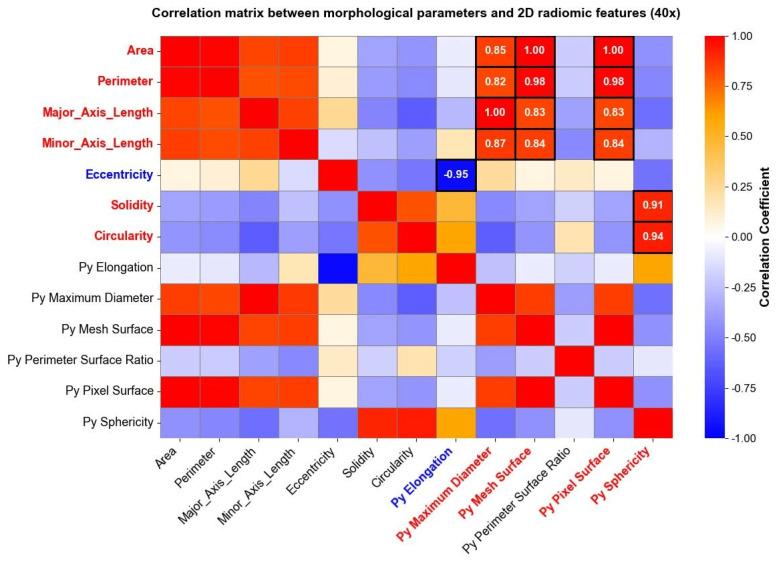
Correlation matrix between the morphological parameters and the “Py” shape 2D radiomic features (10× magnification). The heatmap shows correlation coefficients among variables, ranging from a weak correlation (blue) to a strong correlation (red). High positive (red) and negative (blue) correlations between morphological parameters and shape 2D radiomic features are directly reported as numerical values within the corresponding cells.

Radiomic features were extracted and studied, as well, separately for each level of magnification deriving from the image acquisition process. After computing radiomic features, a correlation matrix was computed to evaluate the relationship between radiomic shape features extracted through Pyradiomics and traditional morphological parameters derived from the shape analysis. The analysis revealed specific associations between shape-based radiomic descriptors and previously established morphological metrics. Figure 6 reports correlation matrices computed for nuclei segmented from 40× magnification images, where high positive and negative correlation coefficients between morphological parameters and shape 2D radiomic features are enhanced through white circles.

**Figure 7:**
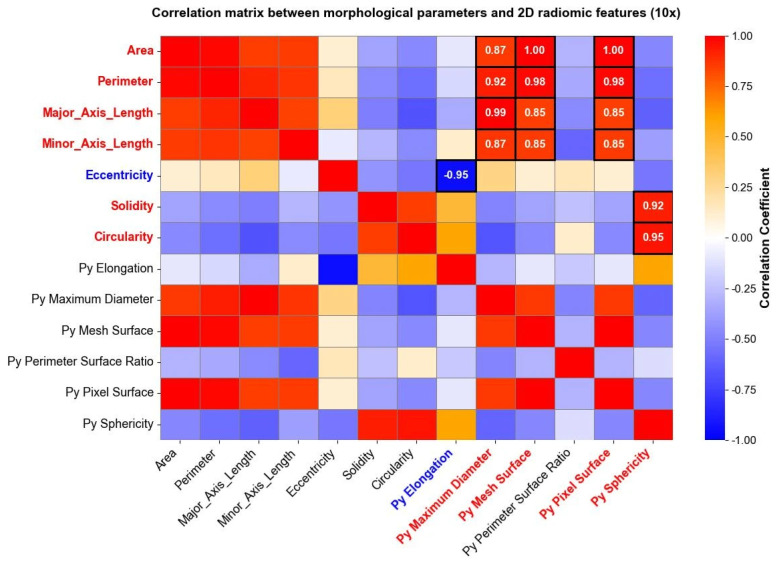
Correlation matrix between the morphological parameters and the “*Py*” shape 2D radiomic features (10× magnification). The heatmap shows correlation coefficients among variables, going from a weak correlation (blue) to a strong correlation (red). High positive (red) and negative (blue) correlations between morphological parameters and shape 2D radiomic features are directly reported as numerical values within the corresponding cells.

The same analysis was carried out for images taken at a 10× magnification level, and the results are reported in Figure 7. Both in Figure 6 and Figure 7, parameters indicated with “Py” header represent radiomic shape 2D features, while the remaining parameters belong to the traditional morphological analysis. Correlation matrices were used solely for exploratory visualization of associations between features, without inferential or hypothesis-testing purposes.

Subsequently, PCA was applied to the extracted radiomic features. 40× magnification and 10× magnification images were analyzed separately. Results from PCA applied to both 40× magnification and 10× images are reported in terms of the scree plot of explained variance (Figure 7) and variable ranking in PCs (Table 3).

Using the “elbow” criterion applied to the scree plot [39], seven principal components were selected for both scree plots as they represented the point where the explained variance began to stabilize, as indicated by the “elbow” observed in Supplementary Figure S1a,b. The percentage of variance explained by each PC is reported in Table 3.

Secondly, through the analysis of each selected PC’s it was possible to determine which features contributed mostly to the determination of selected PCs. In fact, important variables in a component are identified by their high loadings, while unimportant ones are characterized by loadings close to zero [40]. For each PC, the feature with the maximum loading was identified and, subsequently, the features with a loading of at least 95% of the maximum feature’s loading were selected and reported in Table 3.

## 4. Discussion

From a clinical perspective, the proposed cytology-based radiomic analysis is not intended to replace standard diagnostic or follow-up tools such as ultrasound imaging or conventional cytological evaluation, which remain fundamental in the management of benign thyroid nodules. Rather, the present approach should be interpreted as a complementary and hypothesis-generating methodology, potentially integrable at a specific stage of the clinical workflow, namely after benignity has been confirmed by fine-needle aspiration cytology (FNAC) and before radiofrequency ablation (RFA) is performed. This pre-treatment stage represents a delicate step in thyroid nodule management, as clinical decisions may still be influenced by residual cytological heterogeneity and diagnostic uncertainty, even after benign classification [41,42].

In this pre-treatment phase, radiomic features extracted from cytological images may provide additional quantitative descriptors of nuclear heterogeneity and texture, which are not captured by qualitative cytological assessment or ultrasound-derived volumetric measurements. Histopathological studies have shown that thyroid nodules subjected to thermal ablation may exhibit treatment-induced architectural and cytological alterations, which can complicate conventional morphological interpretation and, in some cases, mimic malignant features, thereby requiring careful differential diagnosis and ancillary methods [43].

Within this framework, PCA was used exclusively to explore the internal variance structure of cytological radiomic features, and not as a dimensionality reduction technique aimed at prediction or classification. Notably, several radiomic features were relevant across both magnification levels. For example, original_firstorder_Entropy and original_firstorder_Range contributed to PC1 in both cases, suggesting their independence from image scale. In contrast, other features appeared only in PCs derived from 10× images, suggesting a possible dependence on magnification. Across both magnifications, PC1 and PC2 were dominated by first-order features and texture features deriving from GLCM and GLRLM matrices. In particular, features related to Entropy suggest that complexity and intensity variability in tissues can be interpreted as key aspects for data variance. The presence of original_ngtdm_Coarseness (40×) and original_ngtdm_Strength (10×) further indicates that texture granularity and local variations play an important role in tissue characterization. Morphological features appeared to be particularly relevant in 10× PCs, suggesting that nuclear shape strongly contributes to data variability.

Some shape radiomic features exhibited strong correlations with traditional morphological parameters, indicating that radiomics can capture information related to nuclear geometry. For example, srea was correlated with Py Mesh Surface, perimeter with Py Maximum Diameter and Py Mesh Surface, and circularity and solidity with Py Sphericity. Eccentricity was negatively correlated with Py Elongation. These findings support the feasibility of using radiomics as a complementary tool for quantitative cytological assessment [44].

Overall, radiomic features provide a deeper characterization of tissue texture and intensity patterns beyond traditional morphological measurements. While traditional descriptors such as nuclear circularity, solidity, and eccentricity provide basic assessment, radiomics captures finer spatial and intensity-related information.

In this study, individual nuclei were treated as image-derived observations and not as statistically independent samples. Accordingly, no inferential statistical analysis was performed, and all results should be interpreted as descriptive and exploratory. Due to the limited sample size, no association between cytological radiomic features and VRR can be established. At this stage, the proposed framework is not intended to replace ultrasound or cytological assessment, but rather to complement follow-up evaluation by highlighting cytological heterogeneity that may be associated with different post-RFA trajectories. No predictive model, classification task, or validation strategy was implemented, so no performance metrics were computed. To the best of our knowledge, no previous studies specifically investigated the correlation between traditional morphological parameters and radiomic shape descriptors in thyroid nodules.

The present findings are consistent with existing literature demonstrating the potential of radiomics in histological and cytological image analysis, particularly for the characterization of tissue heterogeneity and nuclear morphology. Previous studies applying radiomic analysis to whole-slide images or segmented nuclei have shown that texture- and intensity-based descriptors can capture information not fully represented by conventional morphological metrics. In line with these observations, first-order and texture features accounted for a substantial proportion of variance in the cytological images analyzed in this study, supporting the relevance of radiomics for quantitative cytological characterization [22,23,45].

Furthermore, the comparison between radiomic shape features and traditional morphological parameters revealed a high degree of correlation between these two feature categories. This finding supports the interpretation of radiomics as a quantitative extension of conventional cytological evaluation rather than a replacement, reinforcing its potential role as a complementary analytical tool for the study of thyroid nodules.

Several limitations should be acknowledged. The limited number of patients reflects the exploratory methodological nature of this study; therefore, no formal sample size calculation was performed, and the findings should be considered preliminary. Approximately 5% of nuclei were not recognized by the segmentation algorithm, likely due to complex cytological regions or limitations in segmentation sensitivity; this issue will be addressed in future work through improved segmentation and validation. Although nuclei segmentation was automated, some parameters required manual adjustment, and the reproducibility of the workflow across different operators was not assessed; future studies will include multi-operator validation. The effect of magnification on feature extraction could not be fully evaluated. Additionally, varying the temporal granularity of follow-up assessments could be explored in future studies to gain further insight into the dynamics of cytological changes and enhance the stability of observation. Despite these constraints, the proposed framework demonstrates feasibility and provides a methodological foundation for more comprehensive future investigations. The present findings should therefore be interpreted as methodological and exploratory, rather than as evidence supporting immediate clinical implementation.

Expanding the patient cohort, increasing the number of cytological images, standardizing magnification levels, and integrating advanced AI-based models may improve interpretability and enable patient stratification based on cytological heterogeneity. Eventually, such developments could inform the future development of predictive models for VRR under controlled and validated conditions.

## 5. Conclusions

In this study, a novel radiomics-based methodological framework for thyroid nodules examination has been presented. The proposed workflow integrates chromatic analysis, automated segmentation, and radiomic features extraction of cytological images, taken at different magnification levels.

Exploratory PCA illustrated that selected radiomic features contribute to the variance structure of cytological images, supporting the potential complementary role of radiomics alongside conventional cytological evaluation.

Although the sample size does not allow clinical conclusions on VRR prediction, this work demonstrates the feasibility of integrating chromatic segmentation, radiomics, and PCA in cytological analysis of thyroid nodules. These findings should be interpreted as methodological and hypothesis-generating, offering a structured foundation for future studies aimed at improving quantitative cytological characterization and supporting translational research.

## Figures and Tables

**Table 1:** Nodule volume and Volume Reduction Rate (VRR) before and after RFA treatment: Volume of each patient’s nodule, before RFA treatment, 30 days and 6 months later, and the respective Volume Reduction Rate (VRR) calculated using the VRR formula. n/a means it is not applicable because the patient was unable to attend, postponing the follow-up visit to the following months.

Patient	Initial Volume	Volume One Month After RFA Treatment	Volume Six Months After RFA Treatment	VRR (1st Follow-Up)	VRR (2nd Follow-Up)
A	5.01 mL	3.56 mL	2.80 mL	29%	44%
B	7.45 mL	5.22 mL	5.70 mL	30%	23%
C	3.05 mL	1.76 mL	n/a	42%	n/a

n/a means it is not applicable.

**Table 2:** Average and standard deviation of nuclear morphological parameters for each patient based on images taken at a 40× magnification level. Average and standard deviation of the morphological parameters (solidity, circularity, and eccentricity) that characterize the nuclei of each patient (Patient A, Patient B, and Patient C). The specific reported parameters were extracted in accordance with the pathologists’ request.

	Patient A		Patient B		Patient C	
	Average	Std	Average	Std	Average	Std
Circularity [0, 1]	0.84	0.08	0.72	0.14	0.77	0.19
Eccentricity [0, 1]	0.56	0.16	0.77	0.14	0.68	0.18
Solidity [0, 1]	0.95	0.02	0.91	0.06	0.90	0.07

**Table 3:** Selection of the most influential radiomic features for each principal component (PC). For each PC, the feature with the highest loading was identified, and features with a loading of at least 95% of the maximum were included. PCA and feature selection were executed separately for 40× and 10× magnification images. The percentage of explained variance for each PC is also reported.

PC (40×)	Features (95% of Maximum Loading)	Explained Variance (%)	PC (10×)	Features (95% of Maximum Loading)	Explained Variance (%)
1	original_firstorder_Entropy original_firstorder_Range	34.33%	1	original_firstorder_Entropy. original_firstorder_MeanAbsoluteDeviation original_firstorder_Range original_glcm_JointEntropy original_glcm_SumEntropy original_glrlm_GrayLevelVariance original_glrlm_HighGrayLevelRunEmphasis original_glszm_HighGrayLevelZoneEmphasis	36.45%
2	original_glcm_Contrast original_glcm_DifferenceAverage original_glcm_Id original_glcm_Idm original_glcm_Idn original_glcm_InverseVariance	17.10%	2	original_gldm_LargeDependenceEmphasis original_glrlm_RunPercentage	21.13%
3	original_glrlm_GrayLevelNonUniformity original_glrlm_RunLengthNonUniformity original_glszm_GrayLevelNonUniformity original_glszm_SizeZoneNonUniformity	10.16%	3	original_shape2D_MeshSurface original_shape2D_PixelSurface original_glrlm_GrayLevelNonUniformity original_glrlm_RunLengthNonUniformity	8.5%
4	original_ngtdm_Coarseness	5.76%	4	original_gldm_LargeDependenceLowGrayLevelEmphasis	5.24%
5	original_firstorder_90Percentile original_firstorder_Mean original_firstorder_RootMeanSquared	4.94%	5	original_glszm_SizeZoneNonUniformityNormalized original_ngtdm_Contrast original_ngtdm_Strength	3.75%
6	original_gldm_LargeDependenceLowGrayLevelEmphasis original_gldm_LowGrayLevelEmphasis	4.68%	6	original_firstorder_90Percentile	3.34%
7	original_gldm_DependenceVariance	3.01%	7	original_firstorder_Kurtosis original_firstorder_Maximum	2.97%

## Data Availability

The partial data used to support the findings of this study are included in the manuscript, and other data used to support the findings of this study are available from the corresponding author upon reasonable request.

## Supplementary Materials

The following supporting information can be downloaded at: https://www.mdpi.com/article/10.3390/bioengineering13020171/s1. Figure S1: Scree plot of PCA applied to features extracted from 40× magnification (**a**) and 10× magnification (**b**) of cytological images. Principal Components (PCs) are represented in blue, while the seven selected components, according to elbow criteria, are represented in orange.

## Abbreviations

The following abbreviations are used in this manuscript:

CAD	Computer-Aided Diagnosis
CNB	Core Needle Biopsy
ECO	Ecography (Thyroid Ultrasound)
EOC	Ente Ospedaliero Cantonale
FNAC	Fine-Needle Aspiration Cytology
FNA	Fine-Needle Aspiration
GLCM	Gray-Level Co-occurrence Matrix
GLDM	Gray-Level Dependence Matrix
GLRLM	Gray-Level Run Length Matrix
GLSZM	Gray-Level Size Zone Matrix
H&E	Hematoxylin and Eosin
HIFU	High-Intensity Focused Ultrasound
IBSI	Imaging Biomarker Standardization Initiative
IQR	Interquartile Range
NGTDM	Neighboring Gray Tone Difference Matrix
PCA	Principal Component Analysis
Py	Prefix for PyRadiomics (i.e., Py Sphericity, Py Mesh Surface)
RFA	Radiofrequency Ablation
RF	Radiofrequency
ROI	Region of Interest
Std	Standard Deviation
TBSRTC	The Bethesda System for Reporting Thyroid Cytopathology
US	Ultrasound
VRR	Volume Reduction Ratio
WSI	Whole Slide Image
