# Supplementary material for: Radiomic Analysis as a Powerful Tool for Cytological Images of Benign Thyroid Nodules Treated by Thermal Radiofrequency Ablation
Source: Bioengineering (Basel). 2026 Jan 30;13(2):171. doi: 10.3390/bioengineering13020171 (PMC12937797; doi:10.3390/bioengineering13020171)
Supplement: Supplementary file 1 [file bioengineering-13-00171-s001.zip › bioengineering-4030693-supplementary.pdf]

SUPPLEMENTARY MATERIAL

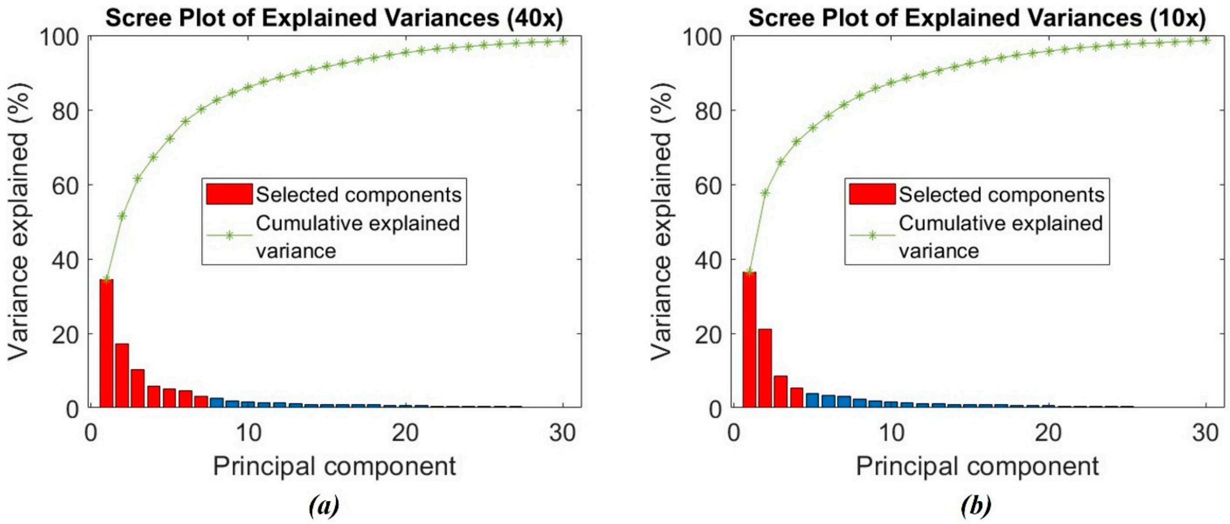

Supplementary Figure S1. Scree plot of PCA applied to features extracted from 40x magnification (**a**) and 10x magnification (**b**) of cytological images. Principal Components (PCs) are represented in blue while the seven selected components, according to elbow criteria, are represented in orange.
